# Features of different severities of reduced working capacity states as an indicator of the self-regulation in students of different courses

**DOI:** 10.1192/j.eurpsy.2022.1585

**Published:** 2022-09-01

**Authors:** M. Abdullaeva, A. Chelak

**Affiliations:** Lomonosov Moscow State University, Psychology, Moscow, Russian Federation

**Keywords:** self-regulation, students, reduced working capacity states, satisfaction with educational activities

## Abstract

**Introduction:**

The relevance of studying of self-regulation styles of young people who face various difficulties at a stage of vocational training has increased in the situation of a pandemic.

**Objectives:**

The purpose of our work is to describe the functional states of students at different stages of education, characterized by different degrees of satisfaction with educational and professional activities and different levels of self-regulation, to substantiate programs of psychological support for students during the formation of their professional identity.

**Methods:**

The sample consisted of 153 students enrolled in 1 and 3 courses in Moscow higher educational institutions. They were asked to fill out a package of methods aimed at diagnosing self-regulation styles (Morosanova, 2020), the severity of states of reduced performance (Leonova, Velichkovskaya, 2002).

**Results:**

Indicators of reduced working capacity among third-year students are statistically significantly higher (p≤0.05) and are in a critical range of severity than among freshmen. Despite a similar level of satisfaction, third-year students demonstrate a whole palette of states of reduced performance, characterized by the experience of monotony of activity with high tension associated with the requirements of the educational situation. The absence of significant differences in the diagnostic indicators of self-regulation obtained in these two groups does not give grounds to assert that the general self-regulation of senior students is higher than that of first-year students.

**Conclusions:**

The data obtained confirm the “painfulness” of the crisis of the “middle” of education and necessarily raise the question of developing a targeted program for mastering the means of conscious self-regulation by students.

**Disclosure:**

No significant relationships.

